# Supplementary material for: Basic emergency obstetric and newborn care service availability and readiness in Nepal: Analysis of the 2015 Nepal Health Facility Survey
Source: PLoS One. 2021 Jul 21;16(7):e0254561. doi: 10.1371/journal.pone.0254561 (PMC8294533; doi:10.1371/journal.pone.0254561)
Supplement: S1 Table — (DOCX) [file pone.0254561.s001.docx]

**S1 Table. Summary of tracer items of each domain and measurement procedure of BEmONC readiness scores**

| **Domain** | **Indicators (Tracer items)** | **Measurement** | **Percent score (%)** | |
| --- | --- | --- | --- | --- |
|  |  |  | **Indicator** | **Domain** |
| **Delivery and new born care index** | | | | |
| Staff and guidelines  (2 indicators) | Guidelines for delivery and newborn care^1^ | Yes  No | 16.67  0.00 | 33.33 |
|  | Staff trained in delivery and newborn care^2^ | Yes  No | 16.67  0.00 |  |
| Equipment  (14 indicators) | Emergency transport | Yes  No | 2.38  0.00 | 33.33 |
|  | Sterilization equipment | Yes  No | 2.38  0.00 |  |
|  | Examination light | Yes  No | 2.38  0.00 |  |
|  | Delivery pack | Yes  No | 2.38  0.00 |  |
|  | Suction apparatus (mucus extractor) | Yes  No | 2.38  0.00 |  |
|  | Manual vacuum extractor | Yes  No | 2.38  0.00 |  |
|  | Vacuum aspirator or D&C kit (with speculum) | Yes  No | 2.38  0.00 |  |
|  | Neonatal bag and mask | Yes  No | 2.38  0.00 |  |
|  | Delivery bed | Yes  No | 2.38  0.00 |  |
|  | Partograph | Yes  No | 2.38  0.00 |  |
|  | Gloves | Yes  No | 2.38  0.00 |  |
|  | Infant weighing scale | Yes  No | 2.38  0.00 |  |
|  | Blood pressure apparatus | Yes  No | 2.38  0.00 |  |
|  | Soap and running water or else alcohol-based hand disinfectant | Yes  No | 2.38  0.00 |  |
| Medicines and commodities  (11 indicators) | Injectable antibiotic | Yes  No | 3.03  0.00 | 33.33 |
|  | Injectable uterotonic | Yes  No | 3.03  0.00 |  |
|  | Injectable magnesium sulphate | Yes  No | 3.03  0.00 |  |
|  | Injectable diazepam | Yes  No | 3.03  0.00 |  |
|  | Intravenous fluids | Yes  No | 3.03  0.00 |  |
|  | Skin disinfectant | Yes  No | 3.03  0.00 |  |
|  | Antibiotic eye ointment | Yes  No | 3.03  0.00 |  |
|  | Chlorhexidine | Yes  No | 3.03  0.00 |  |
|  | Injectable gentamicin | Yes  No | 3.03  0.00 |  |
|  | Injectable ceftriaxone | Yes  No | 3.03  0.00 |  |
|  | Amoxicillin suspension | Yes  No | 3.03  0.00 |  |
| **Total Delivery and newborn care index score** | | | | 100.00 |
| **^1^***The presence of guidelines related to delivery and newborn care (e.g. Nepal Medical Standard Volume III or Reproductive Health Clinical Protocol.*  **^2^** *At least one staff who has received any service-related formal or structured in-service training offered in the last 24 months preceding the assessment.Provider reported receiving skilled birth attendant (SBA) training, advanced skilled birth attendant (ASBA) training, maternal and newborn health updates, training on routine care during labor and normal vaginal delivery, training in active management of the third stage of labor (AMTSL), and any training related to newborn care during the 24 months preceding the survey. The training must have involved structured sessions; it does not include individual instruction that a provider might have received during routine supervision* | | | | |
