# Supplementary material for: Basic emergency obstetric and newborn care service availability and readiness in Nepal: Analysis of the 2015 Nepal Health Facility Survey
Source: PLoS One. 2021 Jul 21;16(7):e0254561. doi: 10.1371/journal.pone.0254561 (PMC8294533; doi:10.1371/journal.pone.0254561)
Supplement: Attachment — Submitted filename: Response to Reviewers_F.docx [file pone.0254561.s003.docx]

Line 26: edit this “We utilizedcross-sectionaldata...”

Line 282: Should be “the” instead of “thee”

Please use “Stata” instead of “STATA.”

Line 296: edit this “UnstandardizedCoefficientthat…”.

There are several places where you need to include a space between a full stop and the beginning of a new sentence (e.g. lines 77, 83, 93).

*R. We edited these typing mistakes. Thank you for highlighting these.*

Please revise the legend of Figure 1 for English grammar.

*R. Revised: BEmONC service readiness index in health facilities by province-wise*

Please indicate the exact p-value instead of p< 0.05. It is fine to write P<0.001.

*R. Indicated. We also added a column of P value in Table 4.*

This study did not assess the quality of care, thus, this statement “The qualities of emergency obstetric services need to be monitored in private hospitals” is not fully supported by the results. Moreover, it is not appropriate to say “qualities”. Please review this also in the conclusion of the main text. The basis for your conclusion on quality of care (which is a much broader concept) is not clear, although this may be a real problem.

*R. The statement was revised:*

*“The private hospitals need to be encouraged for BEmONC service readiness.” in conclusion of abstract.*

*“The availability of the seven signal functions and readiness of BEmONC services was higher in public hospitals compared to private and peripheral public health facilities, indicating the need to improve the quality by increased service readiness in later facility types.” In conclusion of main text.*

You have acknowledged that you did not assess the quality of care (line 440), so addressing the additional comment raised by the second reviewer is at your discretion.  However, “… we did not analyze on process quality…” can be clarified. I guess you meant to say you did not assess the quality of care…

*R. We revised the sentence to make it more clear: “Further, NHFS 2015 did not collect data on health workers’ performance by observation of obstetric service provision, which is an important aspect of quality of care.”*
